# Investigation on the efficiency of *Brucea javanica* oil emulsion injection with chemotherapy for treating malignant pleural effusion: A meta-analysis of randomized controlled trials

**DOI:** 10.3389/fphar.2022.998218

**Published:** 2022-09-16

**Authors:** Rui Shi, Zhishan Wu, Haojia Wang, Jingyuan Zhang, Fanqin Zhang, Antony Stalin, Meilin Chen, Jiaqi Huang, Yiyan Zhai, Qianqian Zhang, Pengyun Liu, Jiarui Wu, Bin Sun, Chunfang Wu

**Affiliations:** ^1^ Department of Clinical Chinese Pharmacy, School of Chinese Materia Medica, Beijing University of Chinese Medicine, Beijing, China; ^2^ Institute of Fundamental and Frontier Sciences, University of Electronic Science and Technology of China, Chengdu, China; ^3^ Jiangsu Jiuxu Pharmaceutical Co. Ltd, Xuzhou, China; ^4^ Beijing Zest Bridge Medical Technology lnc., Beijing, China

**Keywords:** *Brucei javanica* oil emulsion injection, chemotherapy, malignant pleural effusion, meta-analysis., randomzied controlled trials

## Abstract

**Introduction:** Systematic evaluation of the clinical efficacy and safety of Brucea javanica oil emulsion injection (BJOEI) in combination with chemotherapy in the treatment of malignant pleural effusion (MPE).

**Methods:** The study searched CNKI, Wanfang database, VIP database, SinoMed, PubMed, Embase, the Cochrane Library, and the Web of Science database and retrieved randomized controlled trials (RCTs) on the treatment of MPE with BJOEI in combination with chemotherapy from seven electronic databases from inception to 31 March 2022. Meta-analysis and sensitivity analysis were performed using Revman 5.4 and Stata 13.0 software.

**Results:** Ultimately, 30 RCTs with 2035 patients were included, including 1002 cases in the control group and 1033 cases in the treatment group. The results of the meta-analysis showed that the overall efficacy rate of BJOEI combined with chemotherapy was higher in the treatment of MPE compared with chemotherapy alone (*RR* = 1.45, *95%CI*: 1.36–1.54, *p* < 0.00001). And it could improve the Karnofsky (KPS) score (*RR* = 1.54, *95%CI*: 1.41–1.68, *p* < 0.00001), reduce adverse reactions such as fever (*RR* = 0.82, 95%CI:0.60–1.12), chest pain (*RR* = 0.90, *95%CI*: 0.67–1.21), gastrointestinal reactions (*RR* = 0.70, *95%CI*: 0.57–0.87, *p* < 0.005), and leukopenia (*RR* = 0.51, *95%CI*: 0.43–0.61, *p* < 0.00001).

**Conclusion:** BJOEI combined with chemotherapy has better clinical efficacy than chemotherapy alone in the treatment of MPE. It can further improve KPS score, improve patients’ quality of life, and reduce the occurrence of adverse reactions. However, the conclusions of this study need to be confirmed by further randomized, double-blind, controlled trials with large sample size, reasonable design, and strict implementation.

## 1 Introduction

Malignant pleural effusion (MPE), also known as cancerous pleural effusion, refers to pleural effusion caused by metastasis of a primary malignant tumor of the pleura or a malignant tumor in other parts of the pleura. MPE is one of the most common complications in patients with advanced cancer, directly affecting patients’ survival, quality of life, and prognosis. If not treated actively, it can even be life-threatening. Lung and breast cancer are the most common causes, followed by malignant lymphoma and ovarian cancer, as well as metastases and other diseases. The main clinical symptoms are chest tightness, pain, dyspnea, etc. The effect of systemic treatment alone is poor. Therapeutic thoracentesis alone and intercostal thoracic drainage may temporarily relieve dyspnea, but the recurrence rate is high in the short term. The usual clinical treatment modality is to use chemotherapy drugs for local or systemic treatment after thoracic puncture and drainage to relieve symptoms. Chemotherapy controls disease progression by including apoptosis of tumor cells. Cisplatin, bleomycin, and other clinical chemotherapy drugs are also commonly used to treat MPE. However, chemotherapy not only destroys the tumor cells but also impairs normal cellular functions. The adverse reactions are more obvious and often difficult for patients to tolerate. As a result, various complications occur, and the patient’s quality of life is further reduced. Therefore, people are urgently seeking safer and more effective treatment options.

Brucea javanica oil emulsion injection (BJOEI) is a new type of anti-cancer drug consisting of the active ingredients extracted from the mature fruits of the sorrelaceae plant, mainly containing oleic acid and linoleic acid. BJOEI is a non-specific anti-cancer drug that targets the cell cycle. It can inhibit DNA synthesis by inhibiting G0, G1, S, G2 and M phases of tumor cells, and it can also enhance the immune function and hematopoietic function of the human body. When combined with chemotherapy, BJOEI can enhance efficacy and reduce toxicity. In recent years, some studies have shown that BJOEI can reverse the multidrug resistance to chemotherapeutic drugs and also induce apoptosis of tumor cells. Therefore, based on clinical data, this study compared the efficacy and safety of BJOEI in combination with chemotherapy and chemotherapy alone in the treatment of MPE to provide suggestions for doctors to improve clinical treatment plans.

## 2 Methods

### 2.1 Eligibility criteria and exclusion criteria

Studies were considered eligible for inclusion if the following criteria were met.

Study type: Randomized controlled trials (RCTs) of BJOEI combined with a chemotherapy regimen in the treatment of MPE were included. Efficacy results are clear, and data-based outcome indicators are available. RCTs that mention “random” can be included, and their general information should be complete, regardless of the language and country of the literature, regardless of whether concealed allocation and blinding were used. If the RCTs involve multiple treatment regimens simultaneously, only two groups will be selected: Chemotherapy regimens alone and BJOEI in combination with chemotherapy.

Patient: Patients with MPE confirmed to be malignant by pathology, cytology, or imaging examinations, with clinically evident symptoms of pleural effusion such as cough, shortness of breath, chest tightness, and an expected survival period of more than 3 months. There are no restrictions on patient age, gender, race, and cancer type.

Intervention and comparison: After pleural effusion was drained by therapeutic thoracentesis or intercostal pleural drainage, the control group received an intrapleural injection of chemotherapy drugs or systemic chemotherapy (including cisplatin, carboplatin, oxaliplatin, gemcitabine, pemetrexed, bleomycin, mitomycin, and gefitinib); the treatment group received BJOEI in combination with the control group’s pleural injection treatment regimen.

Outcome: All clinical data, including total effective rate, Karnofsky (KPS) score improvement rate, and adverse reactions, can be included. ① Total effective rate: according to the WHO standard, it is divided into complete remission (CR): the effusion disappears, symptoms are relieved and maintained for more than 4 weeks; partial remission (PR): effusion is significantly reduced (more than 50%), symptoms are relieved, and are maintained for 4 weeks or longer; stable disease (SD): less than 50% reduction in pleural effusion, or increase, but not more than 25%; progressive disease (PD): more than 25% increase in pleural effusion or patient death. Total effective rate (%) = (CR + PR)/total number of cases × 100%. In some literature, the efficacy criteria are expressed slightly differently, but they hardly affect the total amount of CR + PR data. The SD data of 11 papers [6,9,15,17–18,22,24,26–28,30] was merged with the PD data, but this did not affect the total efficiency of the calculation. ② Quality of life improvement rate: according to the KPS scoring standard for quality of life, an increase of 20 points after treatment compared with before treatment was considered markedly effective; an increase of 10 points was considered effective; an increase or decrease of <10 points were considered stable; a decrease of more than 10 points was considered a decline. The markedly effective data and effective data give the total improvement rate data. There are eight literatures [3,6–7,15,16,24,26,30] with slightly different KPS scoring standards, of which 4 literatures [6,24,26,30] combined stable data and declining data but did not affect the improvement rate data. ③ Adverse reactions: Fever, chest pain, gastrointestinal reactions, and leukopenia occur more frequently, and forest plots are made separately for analysis.

Documents that meet the following conditions are excluded: ①The article could not be obtained in full text. ②No related outcome. ③the literature with the repeated publication or unknown data.

### 2.2 Search strategy

Use the computer to search CNKI, Wanfang Database, VIP Database, SinoMed, PubMed, Embase, the Cochrane Library, and the Web of Science database. “Brucea javanica oil emulsion” and “Javanica oil emulsion injection” are the names of the drug. “Malignant pleural effusion”, “cancerous pleural effusion”, “cancerous pleural fluid”, etc. are the names of the diseases, and RCTs or randomized controlled trials are the search terms. Furthermore, correlative reference documents were retrieved manually.

#1 “Malignant pleural effusion” [Mesh]
#2 “MPE” [Title/Abstract] OR “Cancerous Pleural Fluid” [Title/Abstract] OR “Cancerous Pleural Effusion” [Title/Abstract]
2#3 #1 OR #
#4 “yadanziyouru zhusheye” [Title/Abstract] OR “yadanziyouru zhusheji” [Title/Abstract]) OR “yadanziyouru injection” [Title/Abstract]) OR “yadanziyouru” [Title/Abstract]) OR “Brucea javanica oil emulsion” [Title/Abstract]) OR “Javanica oil emulsion injection”
4#5 #3 AND #

### 2.3 Literature selection and data extraction

Two researchers independently read the titles and abstracts of the literature and then filtered out duplicates, reviews, pharmacological experiments, and irrelevant articles. The full text of the remaining RCTs was read to assess whether they met the inclusion criteria. If any disagreements occurred, they were resolved by discussion or consultation with a third researcher. After screening, the following information was extracted from the RCTs: ① Basic information of the study (including first author and publication date); ② Study characteristics (including the number of patients in the treatment and control group, sex ratio, mean age, intervention measures and course of treatment); ③ Outcomes; ④ RCTs types and quality assessment factors.

### 2.4 Risk of bias assessment

Two independent researchers assessed the methodological quality according to the Cochrane Risk of Bias Assessment Tool 1.0, and any disagreements were resolved by discussion or consultation with a third researcher. The assessment included the following: ① Sequence generation (selection bias); ② Allocation concealment (selection bias); ③ Blinding of patients and personnel (performance bias); ④ Implementation of blinding of outcome assessors (detection bias); ⑤ Incomplete outcome data (attrition bias); ⑥ Selective outcome reporting (reporting bias); ⑦ Bias from other sources. Each bias includes three judgments: “high risk”, “unclear” and “low risk”. “High risk” means the research used wrong blinding methods, wrong implementation methods, missing data, etc.; “Unclear” means it was not mentioned in the study and cannot be judged; “Low risk” means the implementation method was correct, or did not affect the outcome measurements etc.

### 2.5 Statistical analysis

Revman 5.4 and Stata 13.0 software were used to synthesize and analyze the data. The relative risk (RR) was used as the statistic for the analysis of dichotomous variables of the index effect. 95% confidence intervals (95%CIs) were calculated to indicate the range of results. Chi-square analysis was used to study heterogeneity, and *I* ^*2*^ assessed the magnitude of heterogeneity. For the meta-analysis, the fixed-effects model was used when *p* > 0.1, *I* ^*2*^ < 50%; otherwise, a random-effects model was used. After the results were obtained, *p* ≤ 0.05 indicates that the results are significant; otherwise, the risk difference (RD) is used instead of RR as the indicator effect analysis statistic of the dichotomous variable. If there is no qualitative change in the Meta analysis results, this indicates that the actual results of the RR value are reliable. For publication bias analysis, a funnel plot was drawn, and a sensitivity analysis was performed by Stata 13.0 to assess the stability of the results.

## 3 Results

### 3.1 Literature retrieval and screening result

**FIGURE 1:**
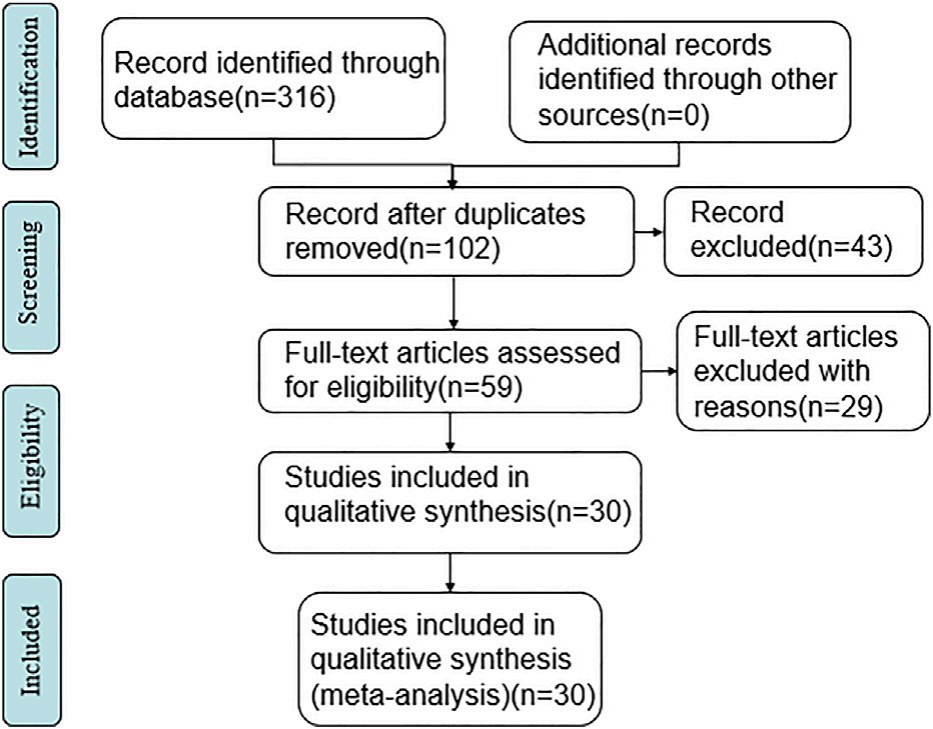
Literature search and screening process.

A total of 316 relevant literatures were detected, and all of them were in Chinese. After deduplication, reading titles, and abstracts to exclude irrelevant literature, there were 60 articles left. Further, read the full text of the literature that may meet the criteria after the initial screening and excluded 29 literature, such as non-RCT studies and data that could not be extracted, 30 RCTs were finally included. The specific search situation is shown in Figure 1.

### 3.2 Characteristics of the included studies

A total of 30 RCTs with 2035 patients were included in this study, 1002 in the control group and 1033 in the treatment group. The details are shown in Table 1.

**TABLE 1:** Included in the list of basic characteristics of the literature.

Study ID	Sample size (EG/CG)	Sex (M/F)	Age (EG/CG)	KPS score	Intervention		Course (d)	Outcomes
					EG	CG		
Gao (2019) ^[1]^	30/30	34/26	53.21 ± 1.12	NR	BJOEI 80–100 ml + CD	DOC 75 mg/m^2^+DDP 20–30 ml/m^2^	21–42	③④⑤⑥
Lu and Zhang. 2018 ^[2]^	45/45	50/40	55.33 ± 11.29/58.49 ± 19.92	NR	BJOEI 30 ml + CD	GEM 1000 mg/m^2^+CBP 30 mg/m^2^;MTA 500 mg/m^2^+CBP 30 mg/m^2^	42	①⑤
Shen. (2017) ^[3]^	40/40	52/28	64.6 ± 4.7/62.5 ± 5.2	≥60	BJOEI 50 ml + DDP	DDP 50 mg/m^2^	28	①②③④⑤⑥
Chen et al. (2016) ^[4]^	19/19	18/20	58.4 ± 6.8/56.9 ± 5.6	NR	BJOEI 50 ml + Gefitinib	GFT 250 mg/d (oral)	90	①
Wang and Song. (2016) ^[5]^	30/30	34/26	63.84 ± 1.59/60.25 ± 1.64	NR	BJOEI 60 ml + DDP	DDP 40 mg/m^2^	14	①②
Fei. (2015) ^[6]^	45/45	59/31	41–79/40–80	≥50	BJOEI 30 ml + BLM	BLM 45 mg	14	①②③④⑤⑥
Chen. (2015) ^[7]^	30/30	35/25	57.2 ± 12.1	≥60	BJOEI 60–90 ml + DDP	DDP 20–30 ml/m^2^	21	①②③④⑤⑥
Huang and Zheng. (2015) ^[8]^	36/36	42/30	59.4 ± 5.3/58.2 ± 4.8	NR	BJOEI 80 ml + BLM	BLM 45 mg	21	①②③⑤⑥
Wang. (2014) ^[9]^	30/30	41/19	31–69/34–66	NR	BJOEI 50 ml + DDP	DDP 3mg/100 ml	28	①
Yang and Sun. (2014) ^[10]^	48/46	60/34	34–67	≥60	BJOEI 80 ml + DDP	DDP 60 mg	28	①②
Zhang et al. (2013) ^[11]^	34/30	52/12	62.5/56	≥50	BJOEI 40–50 ml + DDP	DDP 40–60 ml	28	①④⑤
Gao et al. (2013) ^[12]^	40/40	56/24	67.68 ± 7.68/66.58 ± 9.10	NR	BJOEI 30 ml + CD	DDP 40–60 mg, RIL-2 100 × 10^4^ U	28	①⑤⑥
Wu. (2012) ^[13]^	48/44	58/34	64.5	NR	BJOEI 100 ml + DDP	DDP 60 mg	28	①③④⑤
Liu and Zhang (2012) ^[14]^	32/32	31/33	57.2	NR	BJOEI 100 ml + DDP	DDP 40 ml/m^2^	28	①②③④⑤⑥
Jia et al. (2011) ^[15]^	35/35	38/32	65	NR	BJOEI 60 ml + L-OHP	L-OHP 100 mg/m^2^	28	①⑤
Song et al. (2011) ^[16]^	30/30	35/25	56 ± 11.5	≥60	BJOEI 50 ml + DDP	DDP 40 ml/m^2^	28	①②
Chen (2011) ^[17]^	31/30	27/34	60	≥60	BJOEI 60 ml + DDP	DDP 40–60 mg	28	①③④⑤⑥
Xu (2011) ^[18]^	24/21	-	53.5	≥60	BJOEI 60 ml + MMC	MMC 6 mg	14–21	①②③④⑤⑥
Mo, 2010 ^[19]^	28/28	35/21	50.3/51.8	≥50	BJOEI 60–80 ml + DDP	DDP 80–100 mg	21	①②③④⑤⑥
Fu et al. (2009) ^[20]^	60/60	82/38	51.2	≥50	BJOEI 100 ml + DDP	DDP 40 mg/m^2^	28	①②③④⑤⑥
Wu et al. (2009) ^[21]^	68/55	86/37	65/60	NR	BJOEI 50 ml + DDP 60 mg	DDP 100 mg	28	①⑥
Ji (2009) ^[22]^	30/30	36/24	54.6	≥60	BJOEI 40 ml + DDP	DDP 60 mg	28	①②③⑤
Wang et al. (2007) ^[23]^	35/35	45/25	58	>50	BJOEI 80–100 ml + DDP	DDP 20–30 mg/m^2^	28	①②④⑤⑥
Xie et al. (2007) ^[24]^	23/22	-	53.6/55.2	≥50	BJOEI 60 ml + DDP	DDP 60 mg	28	①②③④⑤⑥
Lei. et al. (2006) ^[25]^	31/30	-	70.03	NR	BJOEI 40–60 ml + DDP	DDP 40–60 mg	21	①②③④⑤⑥
Guo and Wu (2004) ^[26]^	30/30	40/20	59.5	≥40	BJOEI 50 ml + DDP	DDP 150 mg	14	①
Sun et al. (2002) ^[28]^	24/23	30/17	56/52	≥50	BJOEI 80 ml + DDP	DDP 60 mg	21	①②④⑤⑥
Yang and He (2002) ^[27]^	15/15	17/13	60.2/53.4	≥40	BJOEI 50 ml + DDP 60 mg	DDP 40–60 mg	14	①②⑥
You et al. (2001) ^[29]^	30/30	38/22	53.4/60.2	NR	BJOEI 50 ml + DDP 60 mg	DDP 40–60 mg	21	①②
Si et al. (1995) ^[30]^	32/31	41/22	58.3/57.4	≥60	BJOEI 60–80 ml + CD	MMC 10 mg + NFT 1.0 g; MMC 10 mg + ADM 50 mg	28	⑥

CG, control group; EG, experiment group; ①total effective rate; ②KPS, score improvement rate; ③fever rate; ④chest pain; ⑤gastrointestinal reaction; ⑥leukopenia; ADM, doxorubicin; BLM, bleomycin; BJOEI, brucea javanica oil emulsion injection; CBP, carboplatin; DDP, cisplatin; CD, chemotherapeutic drugs; DOC, docetaxel; GEM, gemcitabine; GFT, gefitinib; L-OHP, oxaliplatin; MTA, pemetrexed; MMC, mitomycin; 2:RIL-2, Interlukin2; NFT, nitrofurantion; NR, not report.

### 3.3 Risk of bias assessment

**FIGURE 2:**
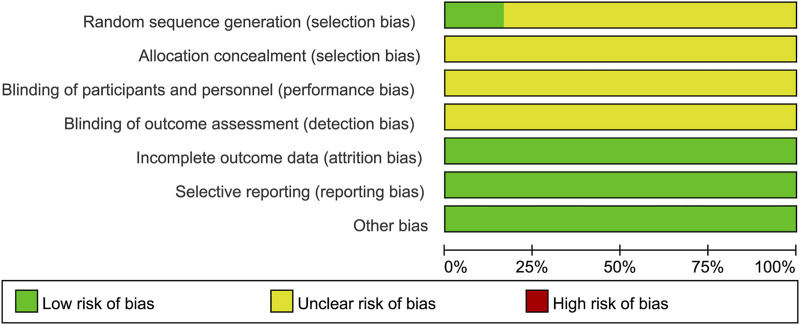
Risk bias evaluation diagram of the included literature.

The 30 included studies had a consistent baseline, and the interventions were parallel. All 30 studies were randomized controlled trials, and all mentioned random assignment. Among the 30 RCTs, one RCT (Lu and Zhang, 2018) used the random sampling method, and three RCTs (Gao et al., 2013; Chen, 2015;, 2019) used the random number table method. The above four RCTs (Gao et al., 2013; Chen, 2015; Lu and Zhang, 2018;, 2019) were rated as “low risk” for selection bias because they generated an adequate random sequence. All studies reported complete outcome data and were rated as “low risk” for attrition bias due to incomplete data. The remaining studies were rated as “unclear” due to insufficient information. The overall situation of quality assessment was as shown in Figure 2.

### 3.4 Outcomes

#### 3.4.1 Clinical total effective rate

**FIGURE 3:**
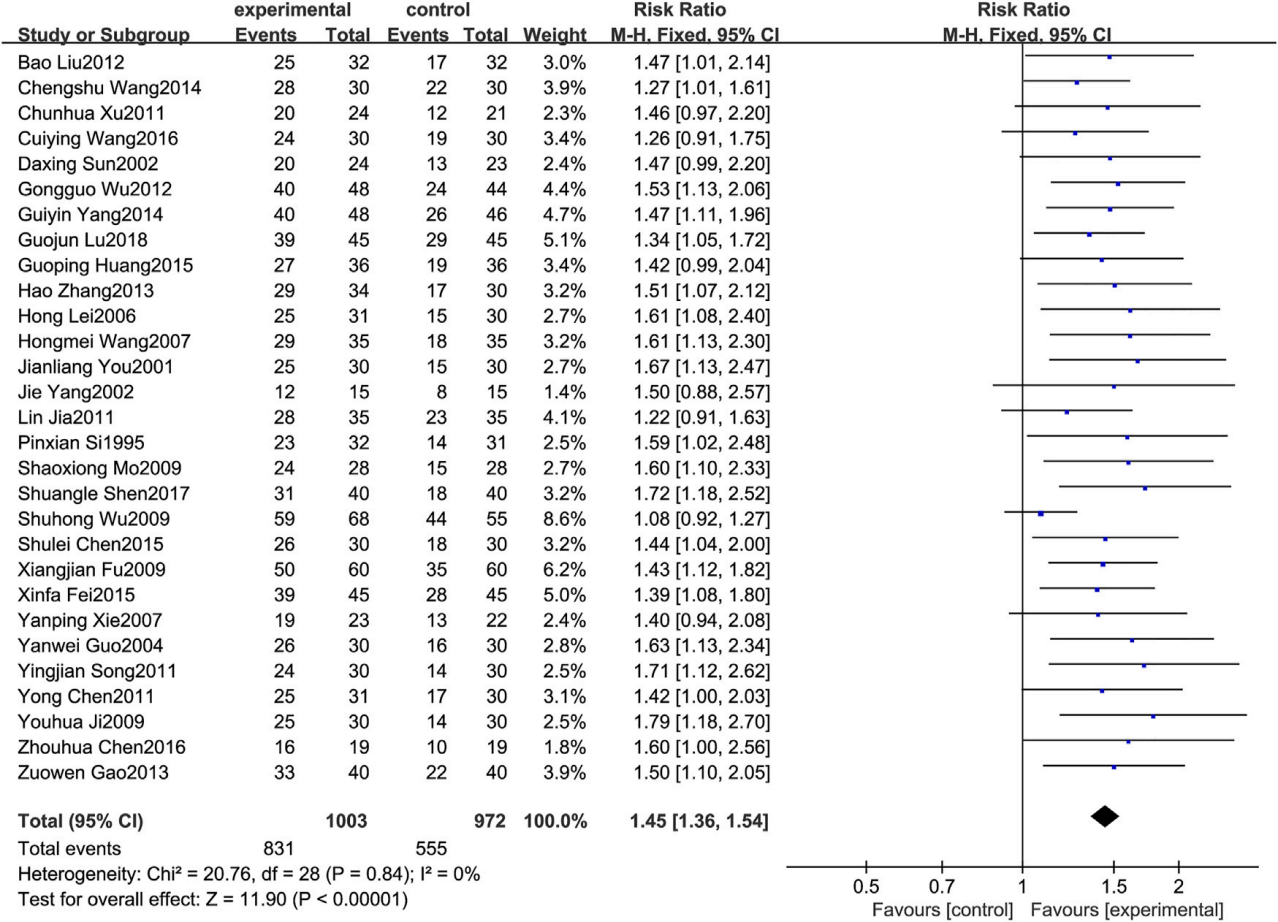
Forest plot of total effective rate.

A total of 29 RCTs [2–30] reported the clinical total effective rate. Since the heterogeneity results showed *p* = 0.84 and *I* ^*2*^ = 0%, the fixed effects model was selected for analysis in this study. The results showed that the total effective rate of the treatment of MPE with BJOEI in combination with chemotherapy drugs was significantly improved compared with using chemotherapy drugs alone, and the difference between the two groups was statistically significant (*RR* = 1.45, *95%CI*:1.36–1.54, *p* < 0.00001), as shown in Figure 3.

#### 3.4.2 KPS score improvement rate

**FIGURE 4:**
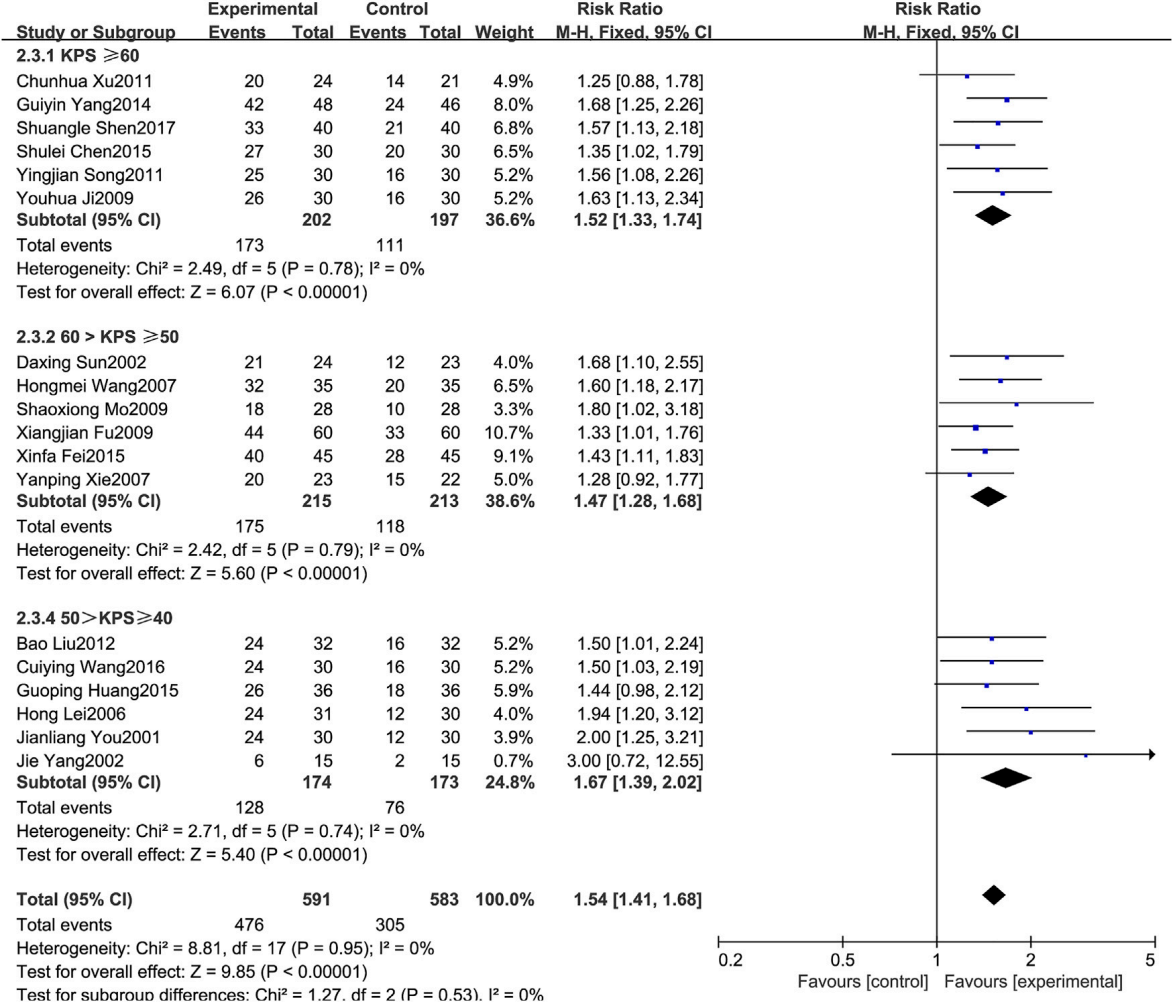
Forest plot of KPS score improvement rate.

A total of 18 RCTs [3,5,6–8,10,14,16,18,20–21,23–26,28–30] reported the KPS score improvement rate. Since the heterogeneity results showed *p* = 0.95 and *I* ^*2*^ = 0%, the fixed effects model was selected for analysis in this study. The results showed that the treatment of MPE with BJOEI in combination with chemotherapeutic drugs could further improve the KPS score and improve the patient’s quality of life compared with the use of chemotherapeutic drugs alone. The difference between the two groups was statistically significant(RR = 1.54, 95%CI: 1.41–1.68, *p* < 0.00001), as shown in Figure 4.

According to the KPS score of the included cases, patients were divided into three groups: KPS score ≥60 points, KPS score ≥50 points, KPS score ≥40 points, and subgroup analysis was performed. There was little difference in the results between the groups, so the results were pooled.

#### 3.4.3 Fever rate

**FIGURE 5:**
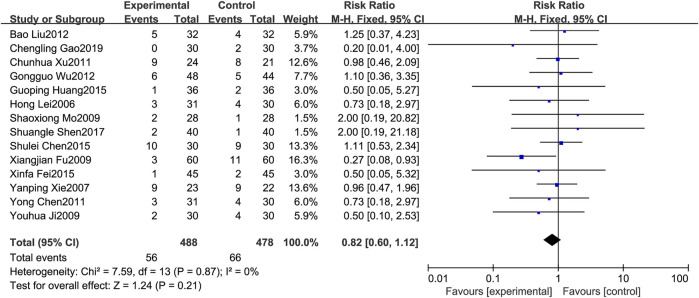
Forest plot of Fever rate.

A total of 14 RCTs [1,3,6–8,13–14,17–18,20–21,23,25–26] reported on fever rate. Since the results of heterogeneity showed *p* = 0.87 and *I* ^*2*^ = 0%, the fixed effects model was selected for analysis in this study. The results showed that the treatment group could reduce the probability of fever to a certain extent compared with the control group. However, it was not statistically significant(RR = 0.82, 95%CI: 0.60–1.12), as shown in Figure 5.

#### 3.4.4 Chest pain

**FIGURE 6:**
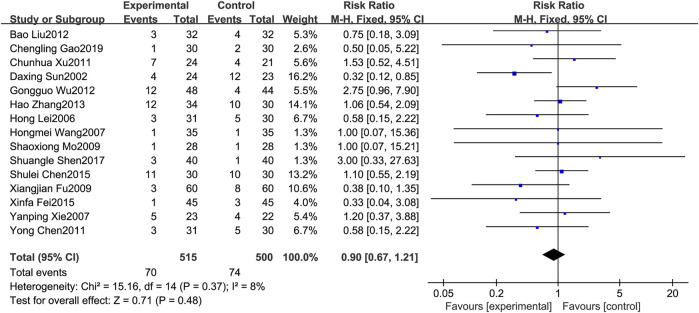
Forest plot of chest pain.

A total of 15 RCTs [1,3,6–7,11,13–14,17–20,23–25,27] reported the chest pain. Since the results of heterogeneity showed *p* = 0.37 and *I* ^*2*^ = 8%, the fixed effects model was selected for analysis in this study. The results showed that the treatment group could reduce the occurrence of chest pain to a certain extent compared with the control group, but it was not statistically significant(RR = 0.90, 95%CI: 0.67–1.21), as shown in Figure 6.

### 3.4.5 Gastrointestinal reactions

**FIGURE 7:**
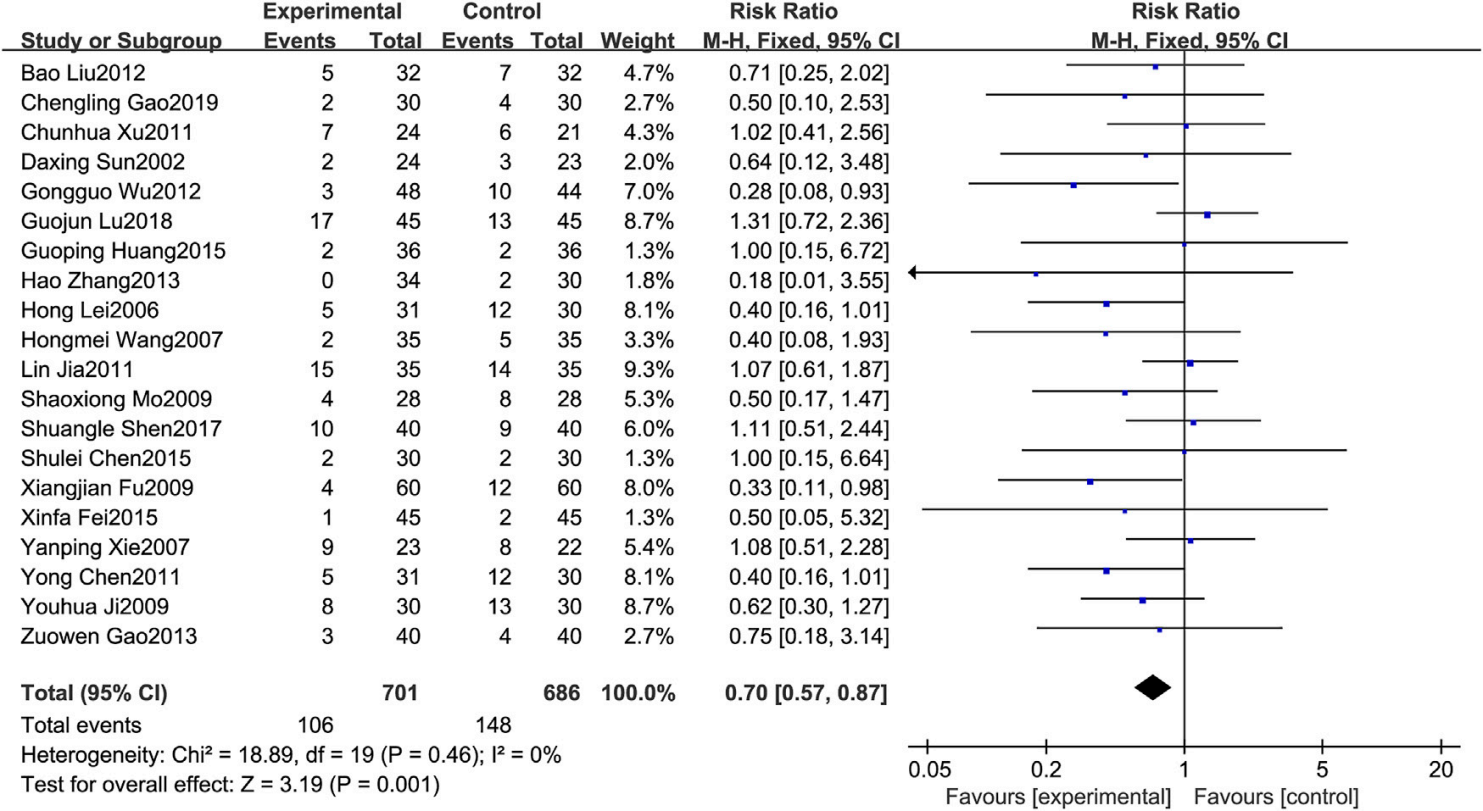
Forest plot of gastrointestinal reaction.

A total of 20 RCTs [1–3,6–8,11–15,17–20,21–25,27] reported on gastrointestinal reaction. Since the results of heterogeneity showed *p* = 0.46 and *I* ^*2*^ = 0%, the fixed effects model was selected for analysis in this study. The results showed that treatment of MPE with BJOEI in combination with chemotherapeutic drugs compared with the use of chemotherapeutic drugs alone, can reduce the probability of gastrointestinal reactions to some extent. The results were statistically significant (RR = 0.70, 95%CI: 0.57–0.87, *p* < 0.005), as shown in Figure 7.

### 3.4.6 Leukopenia

**FIGURE 8:**
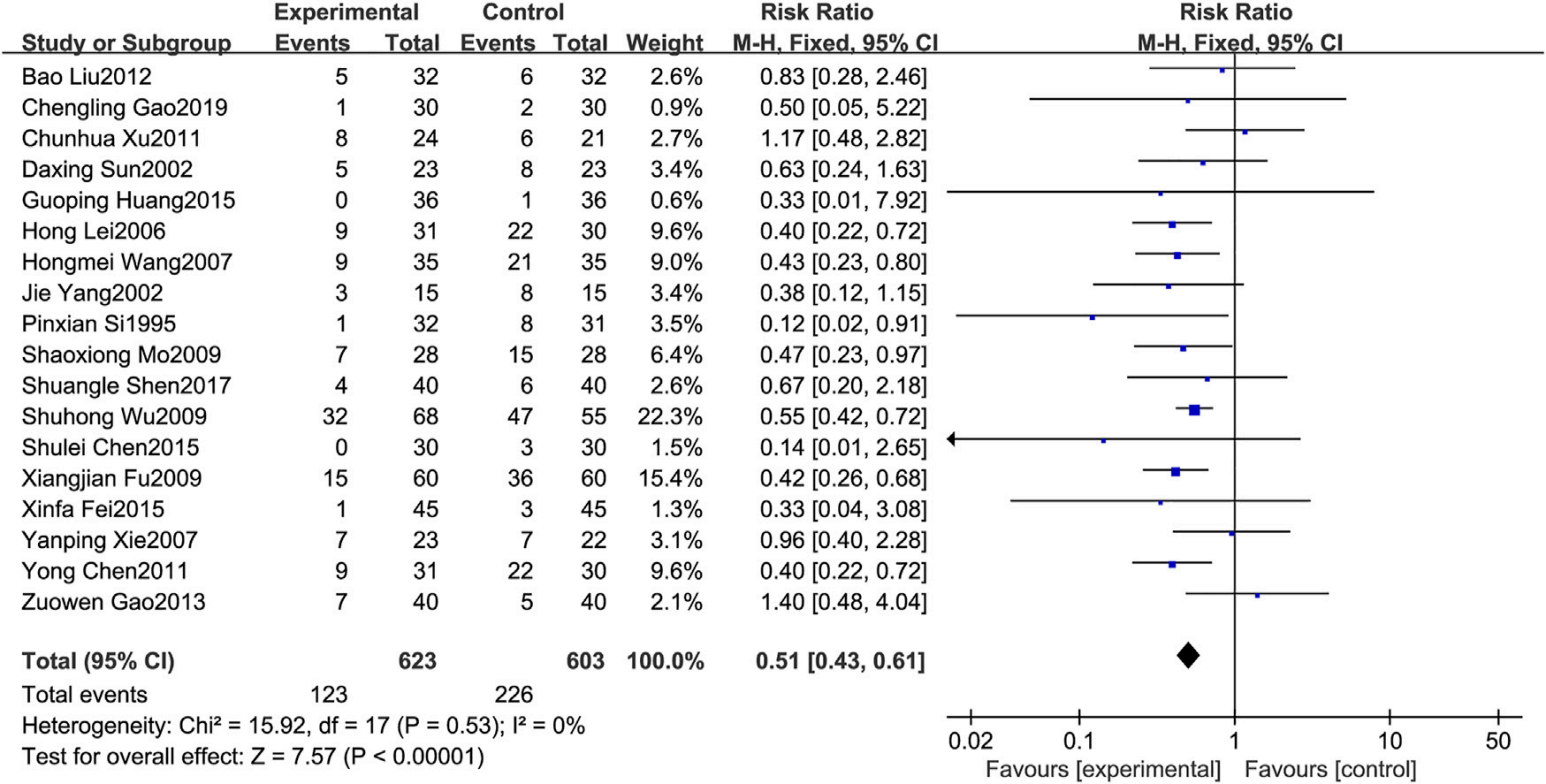
Forest plot of leukopenia.

A total of 18 RCTs [1,3,6–8,12,14,17–21,23–25,27–28,30] reported leukopenia. Since the results of heterogeneity showed *p* = 0.53 and *I* ^*2*^ = 0%, the fixed effects model was selected for analysis in this study. The results showed that treatment of MPE with BJOEI in combination with chemotherapeutic drugs can reduce the incidence of leukopenia compared with the use of chemotherapeutic drugs alone, and the results were statistically significant (*RR* = 0.51, *95%CI*: 0.43–0.61, *p* < 0.00001), as shown in Figure 8.

### 3.5 Safety

Among the 30 RCTs, 23 RCTs recorded adverse reactions, including fever, chest pain, gastrointestinal reactions, leukopenia, liver and kidney damage, hair loss, etc. None of the remaining seven RCTs [4–5,9–10,16,26,29] mentioned adverse reactions.

### 3.6 Sensitivity analysis

**FIGURE 9:**
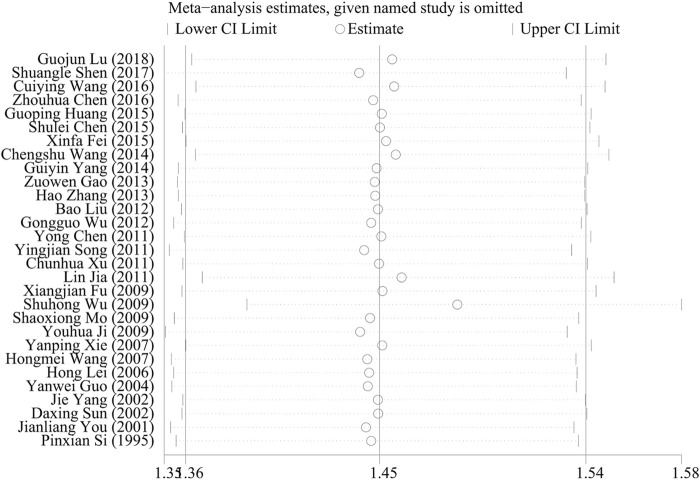
Sensitivity analysis diagram of total effective rate.

Sensitivity analysis was performed using the one-by-one exclusion method for the clinical total effective rate, i.e., one study was excluded each time and the remaining studies were re-analyzed to obtain the RR value and 95%CI and to assess the stability of the results. The results showed that there was no qualitative change in the RR value and 95% CI, indicating that the results of this study were stable, as shown in Figure 9.

### 3.7 Publication bias

**FIGURE 10:**
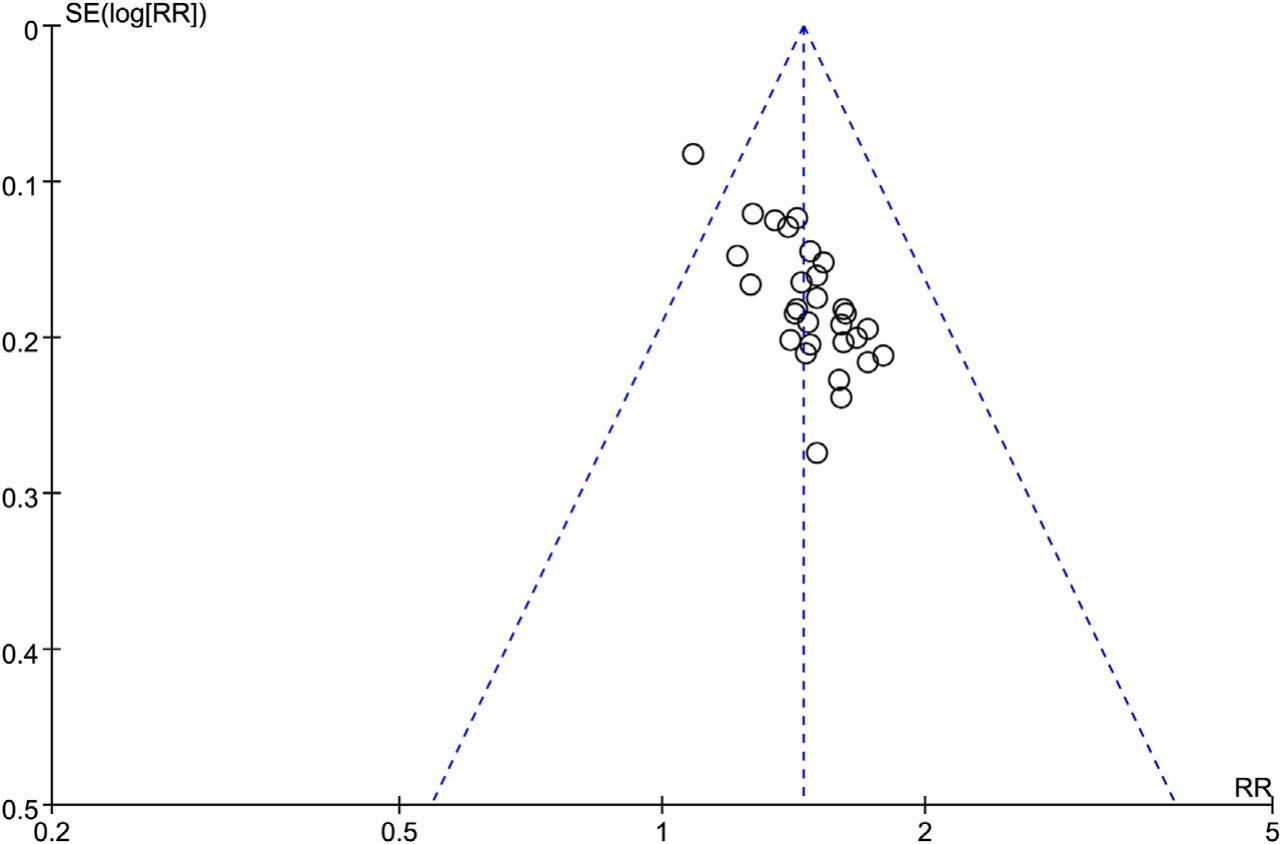
Funnel plot of total clinical effectiveness.

A funnel plot (Figure 10) was used to evaluate the publication bias in the literature for the total effective rate, with the RR value on the horizontal axis and the standard error of the logRR value on the vertical axis. The results showed that the funnel plot was skewed to some degree, the distribution of included studies on the left and right sides of the midline was not completely symmetrical, and one study was outside the auxiliary line, indicating some publication bias.

**FIGURE 11:**
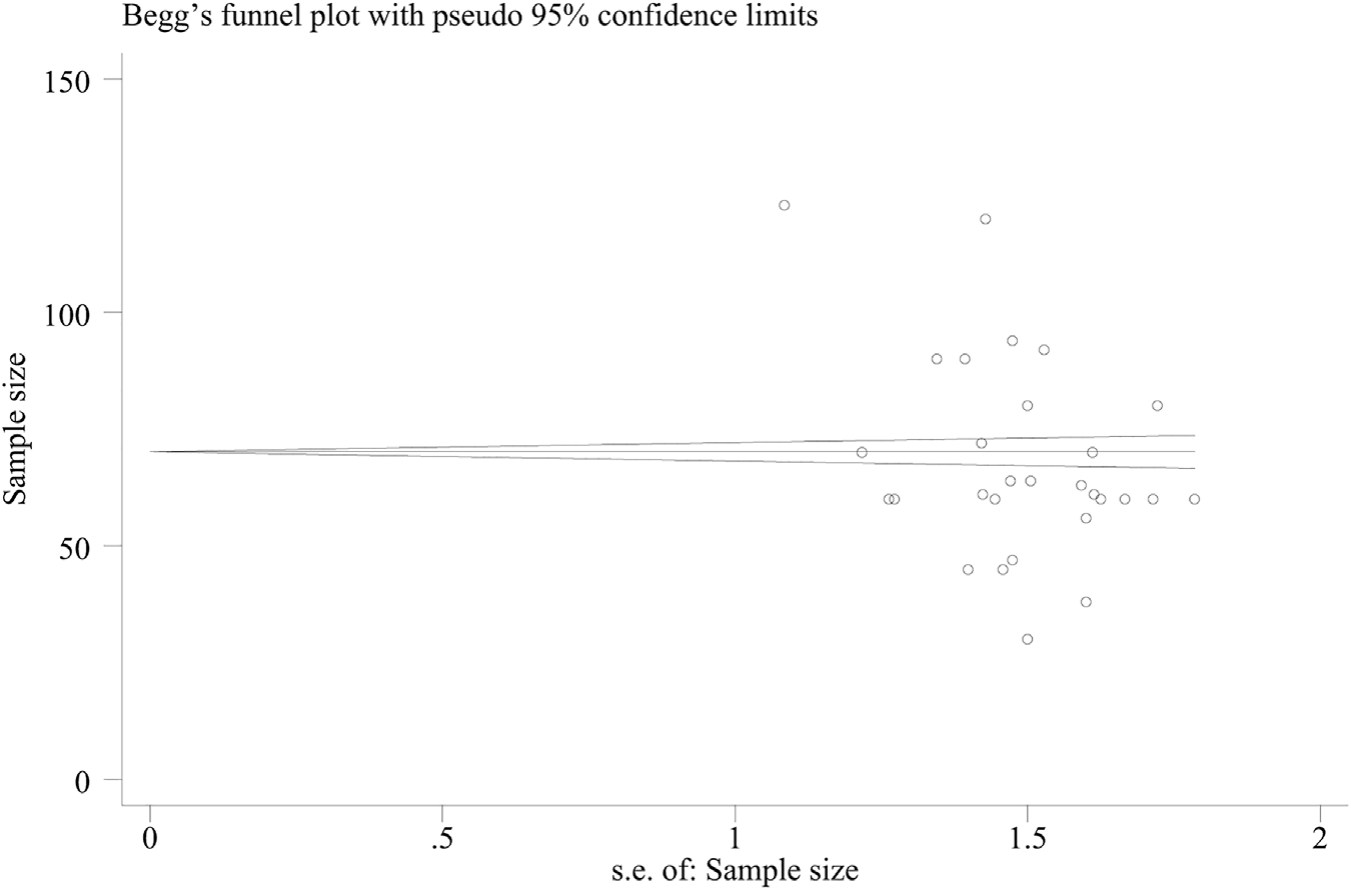
Begg’s funnel plot of total clinical effectiveness.

The Beggar test yielded a value of *p* = 0.019, indicating that there was no significant publication bias (Figure 11).

## 4 Discussion

MPE is a common complication in advanced cancer patients whose effusion is exudate. After the tumor invades the pleura of tumor patients, the permeability increases, a large amount of protein invades the pleural cavity, and the colloid osmotic pressure in the pleural cavity continues to increase. Therefore, when a malignant pleural effusion occurs, the amount of effusion is often large. A large amount of pleural effusion compresses the lungs and affects cardiopulmonary function. Without timely and effective treatment, the pleural effusion will further inhibit the patient’s respiratory and circulatory function, eventually leading to respiratory failure or death.

In the theory of Traditional Chinese medicine, MPE belongs to *xuanyin*, which is caused by the accumulation of phlegm, turbidity, blood stasis and toxins, the blockage of the body’s water channels, and the stagnation of water in the chest and abdomen, which damages the human’s *zhengqi*. BJOEI can protect the *zhengqi*, warm and disperse the knots, and promote the flow of *qi* and blood circulation to treat MPE.

As a new pure anti-cancer drug of Traditional Chinese medicine, BJOEI is known to treat MPE through the following main mechanisms: ① By improving the body’s non-specific immunity, enhancing its ability to generate antibodies, and strengthening the function of phagocytes to achieve the purpose of anti-tumor treatment. ② BJOEI has a specific affinity for tumor cells and can selectively destroy the membrane system of tumor cells. The unsaturated fat in it can directly act on the membrane of the tumor cells, change their surface activity, destroy their biological structure, and finally cause the degeneration and necrosis of tumor cells without affecting the normal cells. ③ Blocks the proliferation of tumor cells by inhibiting their growth and the cell cycle of DNA synthesis. ④ It can specifically inhibit the activity of intracellular POPO II (topoisomerase II), interfere with the normal function of the enzyme, and then cause cell death. ⑤ By stimulating mesothelial cells in the patient’s visceral pleura and parietal pleura to induce chemical pleurisy, the cells proliferate and become fibrotic, resulting in pleural adhesions and pleural atresia, blocking channels for the fluid formation and helping chemotherapeutic drugs to exert their effects. In addition, BJOEI can also reverse tumor resistance to chemotherapy drugs and prolong the application cycle of chemotherapy drugs.

In this study, the average effective rate of the treatment group using BJOEI in combination with chemotherapy in the treatment of MPE was 83.06%, which was a significant improvement over the average effective rate of chemotherapy drugs alone in the control group (57.13%) (*RR* = 1.45, *95%CI*: 1.36–1.54, *p* < 0.00001), and the difference between the two groups was statistically significant. In terms of improving the quality of life, according to the change in KPS score, the average improvement rate in the treatment group was 80.54%, which was also better than the average improvement rate in the control group (52.32%) (*RR* = 1.54, *95%CI*: 1.41–1.68, *p* < 0.00001), the difference was statistically significant.

Regarding safety, Figure 5 shows that the fever rates of the two groups were very close in four studies, and the results of three studies tended to the control group. Figure 6 shows that the chest pain rates of the two groups are very close in four studies, and the results of two studies in the control group are better than those in the treatment group. Figure 7 shows that the incidence of gastrointestinal reactions is very similar between the two groups in the six included studies. Moreover, in one RCT, the results of the control group are better than those of the treatment group. As shown in Figure 8, there are three studies in which the incidence of gastrointestinal reactions is very similar between the two groups. Moreover, the results of the control group are better than those of the treatment group in the two studies. However, specific data analysis cannot prove that the control group intervention is superior to the treatment group. RD was used to re-merge and analyze the data in Figures 5–8, and the same qualitative results as the original effect index RR were obtained, which can be considered more reliable. It was concluded that the incidence of adverse reactions in the treatment group was generally lower than that in the control group. In reducing the incidence of gastrointestinal reactions, the treatment group had a more significant advantage than the control group (*RR* = 0.70, *95%CI*: 0.57–0.87, *p* < 0.05). Similarly, the treatment group also had a significant advantage over the control group in reducing the incidence of leukopenia (*RR* = 0.51, *95%CI*: 0.43–0.61, *p* < 0.00001). In terms of reducing the fever rate and chest pain rate, the treatment group was the same as the control group, and the treatment group had a better effect to a certain extent. However, the meta-analysis results did not show that the difference between the two groups was statistically significant.

At present, there are two meta-analysis articles on the treatment of MPE with BJOEI in combination with chemotherapy, both published in 2014. Zhengbo Hu et al. conducted a meta-analysis on the treatment of MPE with BJOEI in combination with the intrapleural injection of chemotherapeutic drugs. Kongping Wei et al. conducted a meta-analysis on the treatment of MPE with BJOEI in combination with cisplatin alone. In this study, a meta-analysis was performed on the treatment of MPE with BJOEI in combination with chemotherapeutic drugs. These studies did not limit the types of chemotherapeutic drugs, nor were they limited to local injection or systemic chemotherapy. However, the drawback is that some of the included studies did not report the randomization methods and most of the literatures did not mention blinding or allocation concealment.

## 5 Conclusion

In summary, this study verified that BJOEI in combination with chemotherapy drugs could enhance the therapeutic effect of MPE, effectively improve patients’ quality of life, and reduce the toxic and side effects of chemotherapy drugs, which is worth further clinical promotion.

## Abbreviations

95% CI, 95% Credible intervals; BJOEI, Brucea javanica oil emulsion injection; CR, complete remission; KPS, Karnofsky performance status; MPE, malignant pleural effusion; PD, progressive disease; PR, partial remission; PRISMA, the preferred reporting items for systematic reviews and meta-analyses; RCTs, Randomized controlled trials; RD, risk difference; RR, relative risk; SD, stable disease.

## Data Availability

The original contributions presented in the study are included in the article/Supplementary Materials, further inquiries can be directed to the corresponding authors.
