# Supplementary material for: Investigation on the efficiency of Brucea javanica oil emulsion injection with chemotherapy for treating malignant pleural effusion: A meta-analysis of randomized controlled trials
Source: Front Pharmacol. 2022 Sep 16;13:998218. doi: 10.3389/fphar.2022.998218 (PMC9524220; doi:10.3389/fphar.2022.998218)
Supplement: Supplementary file 1 [file DataSheet1.docx]

Supplementary Materials

# File 1: PRISMA checklist for meta-analysis.

**PRISMA checklist for the meta-analysis**

| **Section/topic** | **#** | **Checklist item** | **Reported on page #** |
| --- | --- | --- | --- |
| **TITLE** | | |  |
| Title | 1 | Identify the report as a meta-analysis of randomized controlled trials (or related form of meta-analysis). | 1 |
| **ABSTRACT** | | |  |
| Structured summary | 2 | Provide a structured summary including, as applicable:  Background: main objectives  Methods: data sources; study eligibility criteria, participants, and interventions; study appraisal; and synthesis methods, such as meta-analysis.  Results: number of studies and participants identified; summary estimates with corresponding confidence/credible intervals; a.  Discussion/Conclusions: limitations; conclusions and implications of findings.  Other: primary source of funding; systematic review registration number with registry name. | 2 |
| **INTRODUCTION** | | |  |
| Rationale | 3 | Describe the rationale for the review in the context of what is already known, including mention of why a meta-analysis has been conducted | 2 |
| Objectives | 4 | Provide an explicit statement of questions being addressed with reference to participants, interventions, comparisons, outcomes, and study design (PICOS). | 2-3 |
| **METHODS** | | |  |
| Protocol and registration | 5 | Indicate if a review protocol exists and if and where it can be accessed (e.g., Web address), and, if available, provide registration information including registration number. | / |
| Eligibility criteria | 6 | Specify study characteristics (e.g., PICOS, length of follow-up) and report characteristics (e.g., years considered, language, publication status) used as criteria for eligibility, giving rationale. Clearly describe eligible treatments included in the treatment, and note whether any have been clustered or merged into the same node (with justification). | 3-4 |
| Information sources | 7 | Describe all information sources (e.g., databases with dates of coverage, contact with study authors to identify additional studies) in the search and date last searched. | 4 |
| Search | 8 | Present full electronic search strategy for at least one database, including any limits used, such that it could be repeated. | 4 |
| Study selection | 9 | State the process for selecting studies (i.e., screening, eligibility, included in systematic review, and, if applicable, included in the meta-analysis). | 4-5 |
| Data collection process | 10 | Describe method of data extraction from reports (e.g., piloted forms, independently, in duplicate) and any processes for obtaining and confirming data from investigators. | 4-5 |
| Data items | 11 | List and define all variables for which data were sought (e.g., PICOS, funding sources) and any assumptions and simplifications made. | 4-5 |
| Risk of bias within individual studies | 12 | Describe methods used for assessing risk of bias of individual studies (including specification of whether this was done at the study or outcome level), and how this information is to be used in any data synthesis. | 4-5 |
| Summary measures | 13 | State the principal summary measures (e.g., risk ratio, difference in means). Also describe the use of additional summary measures assessed, such as risk difference (RD) values. | 4-5 |
| Planned methods of analysis | 14 | Describe the methods of handling data. This should include, but not be limited to: Handling of multigroup trials; Selection of variance structure; and Assessment of safety. | 4-5 |
| Risk of bias across studies | 15 | Specify any assessment of risk of bias that may affect the cumulative evidence (e.g., publication bias, selective reporting within studies) | 5 |
| Additional analyses | 16 | Describe methods of additional analyses if done, indicating which were prespecified. This may include, but not be limited to, the following: Sensitivity or subgroup analyses. | 5 |
| **RESULTS** | | |  |
| Study selection | 17 | Give numbers of studies screened, assessed for eligibility, and included in the review, with reasons for exclusions at each stage, ideally with a flow diagram. | 5 |
| Study characteristics | 18 | For each study, present characteristics for which data were extracted (e.g., study size, PICOS, follow-up period) and provide the citations. | 6 |
| Risk of bias within studies | 19 | Present data on risk of bias of each study and, if available, any outcome level assessment. | 6 |
| Results of individual studies | 20 | For all outcomes considered (benefits or harms), present, for each study: 1) simple summary data for each intervention group, and 2) effect estimates and confidence intervals. Modified approaches may be needed to deal with information from larger networks. | 6-7 |
| Synthesis of results | 21 | Present results of each meta-analysis done, including confidence/credible intervals, with full findings presented in an appendix. | 6-7 |
| Exploration for inconsistency | S5 | Describe results from investigations of inconsistency. This may include such information as measures of model fit to compare consistency and inconsistency models, P values from statistical tests, or summary of inconsistency estimates from different parts of the treatment. | / |
| Risk of bias across studies | 22 | Present results of any assessment of risk of bias across studies for the evidence base being studied. | 7 |
| Results of additional analyses | 23 | Give results of additional analyses, if done (e.g., sensitivity or subgroup analyses). | 7-8 |
| **DISCUSSION** | | |  |
| Summary of evidence | 24 | Summarize the main findings, including the strength of evidence for each main outcome; consider their relevance to key groups (e.g., health care providers, researchers, and policymakers). | 8 |
| Limitations | 25 | Discuss limitations at study and outcome level (e.g., risk of bias), and at review level (e.g., incomplete retrieval of identified research, reporting bias). Comment on the validity of the assumptions, such as transitivity and consistency. Comment on any concerns regarding network geometry (e.g., avoidance of certain comparisons). | 9 |
| Conclusions | 26 | Provide a general interpretation of the results in the context of other evidence, and implications for future research. | 910 |
| **FUNDING** | | |  |
| Funding | 27 | Describe sources of funding for the systematic review and other support (e.g., supply of data); role of funders for the systematic review. This should also include information regarding whether funding has been received from manufacturers of treatments in the network and/or whether some of the authors are content experts with professional conflicts of interest that could affect use of treatments in the network. | 10 |

# File 2: Search strategy for meta-analysis

## Search strategy of Brucea javanica oil emulsion injections.

| 序号 | 注射剂名称 | 注射液英文检索词 | 中文检索词 |
| --- | --- | --- | --- |
| 1 | 鸦胆子油乳注射液 | yadanziyouru zhusheye | 鸦胆子油乳注射液OR鸦胆子油乳注射剂OR鸦乳注射液OR鸦乳 |

## Search strategy of Pubmed.

#1 “Malignant pleural effusion” [Mesh]

#2 “MPE” [Title/Abstract]
#3 Cancerous Pleural Fluid [Title/Abstract]
#4 Cancerous Pleural Fluid [Title/Abstract]
#5 Cancerous Pleural Effusion [Title/Abstract]
#6 yadanziyouru zhusheye [Title/Abstract]
#7 yadanziyouru zhusheji [Title/Abstract]
#8 yadanziyouru injection [Title/Abstract]
#9 yadanziyouru [Title/Abstract]
#10 Brucea javanica oil emulsion [Title/Abstract]
#11 Javanica oil emulsion injection [Title/Abstract]
#12 #1 OR #2 OR #3 OR #4 OR #5 OR #6 OR #7 OR #8 OR #9 OR #10 OR #11

## Search strategy of CNKI.

#1 “恶性胸腔积液” [Mesh]

#2 “癌性胸水” [Title/Abstract]
#3 “恶性胸水” [Title/Abstract]
#4 “癌性胸液” [Title/Abstract]
#5 “鸦胆子油乳” [Title/Abstract]
#6 “鸦胆子油乳注射液” [Title/Abstract]
#7 “鸦胆子油乳注射剂” [Title/Abstract]
#8 “鸦乳” [Title/Abstract]
#9 “鸭胆子油乳” [Title/Abstract]
#10 “化疗” [Title/Abstract]
#11 #1 OR #2 OR #3 OR #4 OR #5 OR #6 OR #7 OR #8 OR #9 OR #10 OR

# File 3: Detailed information on included BJOEI

|  | **Name of injection** | **Source** | **Species /Raw materials** | **Botanical plant names** | **Function** | **Indication** | **Quality control reported? (Y/N)** |
| --- | --- | --- | --- | --- | --- | --- | --- |
| 1 | Brucea javanica oil emulsion injections | Jiangsu Jiuxu Pharmaceutical Co., Ltd | Refined Brucea Javanic Oil、 Soybean phospholipid、glycerol、 | *Brucea javanica (L.) Merr* | Clearing heat and detoxifying, dissipating blood stasis and removing knots | Malignant pleural effusion, lung cancer, brain metastasis, etc, etc. | Y-National Pharmaceutical Standard Z19993152 |
